# Beyond Continuity: A Binary‐Outcome Cross‐Lagged Panel Model of Ikigai and Social Interaction Among Community‐Dwelling Older Adults

**DOI:** 10.1111/ggi.70755

**Published:** 2026-08-05

**Authors:** Yu Pei, Aki Yazawa, Chie Nitta, Tetsuhiro Maeno, Kumi Watanabe Miura, Yuko Sawada, Akihiro Kakuda, Tokie Anme

**Affiliations:** ^1^ Graduate School of Comprehensive Human Sciences University of Tsukuba Ibaraki Japan; ^2^ Department of Primary Care and Medical Education, Institute of Medicine University of Tsukuba Ibaraki Japan; ^3^ National Hospital Organization Kurihama Medical and Addiction Center Yokosuka Japan; ^4^ Japan Society for the Promotion of Science (JSPS) Tokyo Japan; ^5^ RIKEN Center for Advanced Intelligence Project Tokyo Japan; ^6^ Department of Physical Therapy Morinomiya University of Medical Sciences Osaka Japan; ^7^ International Community Care and Lifespan Development, Empowerment Sciences, Institute of Medicine University of Tsukuba Ibaraki Japan

**Keywords:** cross‐lagged panel model, Ikigai, longitudinal study, older adults, social interaction

## Abstract

**Aim:**

To examine the longitudinal and potentially bidirectional association between Ikigai and social interaction among community‐dwelling older adults, and to evaluate the feasibility of a binary‐outcome cross‐lagged panel model (CLPM).

**Methods:**

This study analyzed data from the Community Empowerment and Care (CEC) cohort in T‐village in central Japan. A total of 504 participants aged ≥ 65 years who took part in both the 2017 and 2020 surveys were analyzed. Ikigai was assessed by a self‐reported question. Social interaction was measured with the Index of Social Interaction (ISI). A two‐wave CLPM with binary outcomes was modeled using the weighted least squares mean and variance adjusted (WLSMV) estimator in Mplus 8.7, and baseline‐adjusted logistic regression analyses were additionally conducted using SPSS 30.0. Covariates assessed at baseline included age, gender, subjective economic status, depressive symptoms, subjective cognitive complaints, negative life events (NLE), chronic diseases, smoking behavior, drinking behavior, social interaction, and Ikigai.

**Results:**

In the CLPM, richer social interaction in 2017 significantly predicted Ikigai in 2020 (*β* = 0.165, *p* = 0.006), whereas the reverse pathway from baseline Ikigai to subsequent social interaction did not reach conventional statistical significance (*β* = 0.102, *p* = 0.060). Autoregressive effects for both Ikigai and social interaction were substantial, and overall model fit indices indicated an acceptable fit.

**Conclusions:**

Over 3 years, richer social interaction prospectively supported Ikigai, while Ikigai alone did not translate into greater subsequent interaction once temporal dependencies were modeled. Binary‐outcome CLPM proved feasible and informative for examining categorically measured psychosocial constructs in aging research. Interventions that expand opportunities for meaningful social interaction may help sustain Ikigai in later life.

## Graphical abstract

Using a binary‐outcome cross‐lagged panel model, this study examines the longitudinal association between social interaction and Ikigai among Japanese older adults. Richer social interaction predicted subsequent Ikigai, whereas the reverse pathway did not reach conventional statistical significance in the cross‐lagged model, highlighting the potential role of social interaction in later‐life meaning‐oriented well‐being.

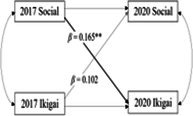

## 1 Introduction

Ikigai, as a term originating from Japanese culture, is a concept that encompasses diverse interpretations reflecting its multifaceted nature. Mathews [1] defines Ikigai as “life worth living,” while Hasegawa et al. [2] describe it as “meaning in life.” Kamiya [3] offers additional perspectives, characterizing Ikigai as both “purpose in life” and “reason for living.” In a broad sense, Ikigai can be conceptualized as the foundation of an individual's survival and the driving force that imbues life with purpose and direction [1]. Furthermore, lexicographical definitions highlight Ikigai as both “enjoying the happiness and contentment of life” and “understanding the significance and worth of one's own existence” [4]. Empirical studies have shown that having Ikigai is associated with a range of positive health outcomes. The Japan Collaborative Cohort Study demonstrated that individuals with Ikigai exhibited a significantly lower risk of all‐cause mortality [5]. Longitudinal evidence from a large‐scale outcome‐wide analysis among older adults in Japan further reported that Ikigai predicted a reduced incidence of functional disability, dementia, and depressive symptoms, as well as higher subjective well‐being and life satisfaction [6]. Cross‐sectional research also supports the link between Ikigai and better quality of life, suggesting that Ikigai may serve as a protective psychosocial resource [7]. Collectively, these studies underscore Ikigai as a key determinant of physical, cognitive, and emotional health among older adults.

Social interaction is commonly defined as interpersonal behaviors and relationships through which individuals fulfill personal needs, and more broadly, the extent of engagement with one's environment [8]. It is increasingly understood as a dynamic and multidimensional process embedded in daily life, encompassing not only direct interpersonal exchanges but also the functional and motivational capacities that enable older adults to initiate and sustain engagement with others and with the surrounding environment over time [9]. Social interaction is associated with both physical [10] and mental health [11], while poor or limited interaction is linked to functional decline, higher mortality [12], dementia [13], and depressive symptoms [14].

A substantial body of longitudinal research has demonstrated that various indicators of social relationships, such as social interaction, social participation [15], social support [16], loneliness [17], and social isolation [9], are associated with mental health outcomes among older adults, encompassing both negative indicators such as depressive symptoms and anxiety [18], and positive indicators such as happiness [19] and life satisfaction [20]. Some studies have further applied longitudinal structural equation modeling approaches, including the cross‐lagged panel model (CLPM) [10, 15, 17], to examine temporal ordering and dynamic interrelations between social relationship indicators and mental health outcomes among older adults. For example, a CLPM analysis in a nationally representative cohort of British adults suggested bidirectional associations between social isolation and depressive symptoms over time [10]. Cross‐lagged findings for loneliness and depressive symptoms have also indicated temporal links, although the strength and symmetry of reciprocal paths may vary across studies and model specifications [21]. Within this broader framework, Ikigai, as a culturally grounded positive psychological concept, is closely related to various forms of social interaction with accumulating evidence. Previous studies have reported that engagement in community‐based activities [22], involvement in volunteer programs [23], and regular and sustained social participation [24] are associated with a higher likelihood of reporting Ikigai. However, despite these consistent associations, most existing studies have relied on cross‐sectional designs, leaving the temporal direction of the relationship between social interaction and Ikigai unresolved. Although a small number of longitudinal studies exist, they have typically focused on unidirectional pathways and have not formally tested reciprocal dynamics between these constructs [25].

Moreover, an additional methodological gap exists in that both Ikigai and social interaction are sometimes measured as categorical variables in population‐based surveys, whereas the conventional CLPM was originally developed for continuous outcomes and relies on assumptions of linearity and normally distributed residuals. When applied to categorical measures, these assumptions may be violated, potentially leading to biased parameter estimates or limited interpretability of cross‐lagged effects [26]. Categorical measures often reflect changes in underlying states rather than incremental changes in magnitude, which are not adequately captured by traditional continuous‐variable formulations. Recent methodological advances have extended CLPM to accommodate categorical outcomes through latent response modeling frameworks [27]. The weighted least squares mean and variance adjusted (WLSMV) estimator in Mplus treats observed categorical variables as manifestations of underlying continuous latent propensities, with category membership determined by whether these propensities exceed specific thresholds. By modeling categorical outcomes in this way, WLSMV enables the examination of longitudinal and reciprocal associations without imposing inappropriate continuity or linearity assumptions on the observed data [28].

In light of these gaps, this study aims to contribute both substantive and methodological insights. Using longitudinal data from a community‐based cohort of Japanese older adults, we examine whether richer social interaction at baseline predicts subsequent Ikigai and whether reciprocal effects are supported over time. Methodologically, we apply a binary‐outcome CLPM using the WLSMV estimator and compare the findings with those obtained from conventional logistic regression, thereby illustrating the feasibility and added value of CLPM for categorical outcomes.

## 2 Materials and Methods

### 2.1 Study Design

This cohort study utilized data from the “Community Empowerment and Care for Well‐being and Healthy Longevity: Evidence from a Cohort Study” (CEC). The CEC is a population‐based survey conducted every 3 years since 1991 in T‐village in central Japan, ranging from infants to older adults and still ongoing. Questionnaires were administered in paper form as self‐reports. In 2017, they were distributed and collected by volunteers recruited by the local government through the drop‐off/pick‐up method, whereas in 2020, the follow‐up survey was conducted immediately after Japan's first COVID‐19 state of emergency, adopting alternative non‐contact distribution and collection procedures. Although Ikigai and social interaction have been measured since earlier waves, including the survey in 2014 would have substantially reduced the analytic sample due to attrition, while the survey in 2023 revised the Ikigai item. Therefore, the present study focused on waves in 2017 and 2020, which provided the most recent and appropriate data for conducting the CLPM. This study was approved by the Ethics Review Committee of the University of Tsukuba (certification No. 1331‐5).

### 2.2 Participants

**FIGURE 1:**
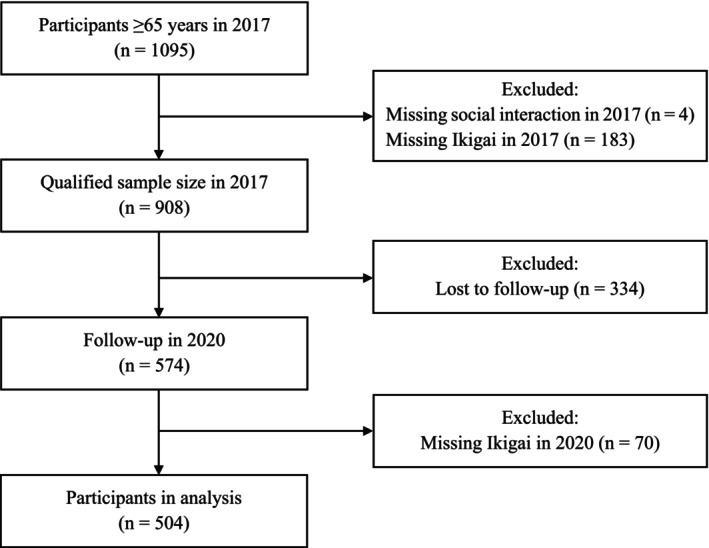
Flowchart of the study participants.

The inclusion criterion for this study was participants aged ≥ 65 years as of 2017. In that year, a total of 1095 individuals met the inclusion criterion and participated in the survey. There were 908 participants who provided responses to the question of Ikigai and at least one item of the Index of Social Interaction (ISI) among them. After excluding 334 individuals who were lost to follow‐up, the sample size of follow‐up in 2020 was 574. Then 70 participants with missing data of Ikigai in 2020 were excluded, and 504 individuals were ultimately included in the analysis (as shown in Figure 1).

### 2.3 Measures

#### 2.3.1 Social Interaction

The Index of Social Interaction (ISI) was employed to appraise participants' social interaction [29]. Reflecting the multidimensional nature of social interaction, the ISI operationalizes this construct through multiple domains capturing various aspects of daily social functioning and engagement among community‐dwelling older adults. The ISI consists of five dimensions, including independence, social curiosity, interaction, participation in society, and feelings of safety, comprising a total of 18 items. The independence dimension is intended to capture the functional and motivational capacities that enable older adults to initiate and sustain engagement with others and with their surrounding environment. In this sense, independence is conceptualized as an enabling condition for social interaction within the conceptualization adopted in this study. For each item, the response of “always/often/sometimes” was scored as 1 and “rarely” was scored as 0, with total scores ranging from 0 to 18 [30]. Although the ISI yields a total score on a continuous scale, the distribution of ISI scores in both 2017 and 2020 was highly skewed toward the upper end, with the majority of participants scoring 17 or 18 and relatively few observations in the lower range (as shown in Appendix S1 and Appendix S2). Under such conditions, treating the ISI as a continuous variable may lead to unstable estimates and reduced interpretability. Therefore, following previous research [31] and based on the sample‐specific median distribution, a cutoff value of 17 was adopted in this study. Individuals scoring ≥ 17 were classified as “rich social interaction,” and those scoring < 17 as “not rich social interaction.” Internal consistency of the ISI subscales has been reported to be acceptable, with Cronbach's alpha values ranging from 0.78 to 0.81 [32]. In addition, evidence for the construct and predictive validity of the ISI has been reported in research of older adults, showing expected associations with relevant health and psychosocial outcomes [33]. Item details are provided in Appendix S3.

#### 2.3.2 Ikigai

Ikigai was assessed using a single self‐reported question, “Do you have any Ikigai in life?” The response options were limited to “yes” and “could not think of any.” Participants who selected “yes” were classified as having Ikigai, whereas those who selected “could not think of any” were classified as not having Ikigai. It should be noted that Ikigai was operationalized as a binary construct by design, rather than through analytic dichotomization, reflecting a common approach in Japanese population‐based research. The question has been used by the Japanese Cabinet Office in national surveys addressing policies for an aging society [34] and has been adopted in previous epidemiological studies as a validated measure of the presence or absence of Ikigai in Japan [35].

#### 2.3.3 Covariates

Age, gender, subjective economic status, depressive symptoms, subjective cognitive complaints, negative life events (NLE), chronic diseases, smoking behavior, drinking behavior, baseline social interaction, and baseline Ikigai [36] were included as covariates to control for potential confounding effects on the association between Ikigai and social interaction. All covariates were categorical variables and gathered from self‐reported questionnaires at baseline. Detailed classifications are shown in Appendix S4.

### 2.4 Statistical Analysis

First, descriptive statistics were employed to summarize the baseline demographic characteristics of participants. Second, the CLPM was applied to examine the temporal ordering and potential reciprocal associations between social interaction and Ikigai across the two waves. Covariates measured at baseline were included as exogenous predictors in the CLPM and were specified to predict the follow‐up measures of Ikigai and social interaction, thereby adjusting the estimated cross‐lagged associations for potential confounding. Although CLPM has traditionally been applied to continuous outcomes, categorical variables can also be modeled as dependent variables within this framework [26]. Methodological advances have extended autoregressive and cross‐lagged models to non‐commensurate and categorical outcomes, demonstrating their feasibility and interpretability [28]. Most recently, Muthén et al. [27] provided detailed illustrations of the CLPM with binary and ordinal outcomes using the weighted least squares mean and variance adjusted (WLSMV) estimator in Mplus, thereby offering concrete guidance for the modeling strategy employed in this study. The practical application of categorical‐outcome CLPM has been supported in recent empirical research across diverse populations and health domains [37, 38]. In line with this methodological and empirical precedent, the present study applies CLPM with binary outcomes using WLSMV estimation in Mplus, thereby providing a reproducible framework for other longitudinal datasets with categorical outcomes. In terms of design, the present study employed a two‐wave CLPM using data from 2017 and 2020. Although many methodological discussions recommend three or more waves to fully capture developmental processes, prior research has demonstrated that two‐wave CLPM can still provide valid and interpretable insights into temporal precedence and bidirectional associations [39, 40]. Accordingly, the current two‐wave design was considered appropriate and feasible given the available data. In addition, to address potential changes in health status and life circumstances during the follow‐up period, a sensitivity analysis was conducted to assess the robustness of the CLPM findings. Changes in NLE and functional status, operationalized as instrumental activities of daily living (IADL), were included as additional covariates predicting follow‐up Ikigai and social interaction. As a supplementary analysis, binary logistic regression analyses were conducted to examine temporally ordered associations between baseline social interaction and subsequent Ikigai, as well as between baseline Ikigai and subsequent social interaction, thereby providing complementary evidence to the CLPM findings.

The degree of model fit was evaluated using multiple indices, including the Comparative Fit Index (CFI), Tucker‐Lewis Index (TLI), Root Mean Square Error of Approximation (RMSEA), and Standardized Root Mean Square Residual (SRMR). Based on conventional benchmarks, RMSEA ≤ 0.06 and SRMR ≤ 0.08 were considered indicative of good fit, whereas CFI and TLI ≥ 0.90 were generally considered acceptable and values ≥ 0.95 indicative of good model fit [41, 42]. Overall model fit was interpreted based on a combination of fit indices rather than strict adherence to a single cutoff, particularly in models with increased complexity and multiple covariates [43]. All coefficients reported were standardized. In addition, missing data were handled with multiple imputation using the Fully Conditional Specification (FCS) method, generating 50 datasets that were analyzed independently and pooled using Rubin's rules. All analyses were conducted using IBM SPSS Statistics Version 30.0 and Mplus Version 8.7, with the significance level set at 0.05.

## 3 Results

Table 1 presents the baseline descriptive statistics of the participants' demographic characteristics. Among the participants, the majority (65.7%) were aged 65–74 years, while 34.3% were ≥ 75 years. Gender distribution revealed a slightly higher proportion of females (53.4%) compared to males (46.6%). Regarding subjective economic status, 21.2% of participants reported being economically disadvantaged. With respect to psychological and cognitive characteristics, 30.2% of participants presented depressive symptoms, and 53.8% presented subjective cognitive complaints. In the case of the NLE, 74.0% experienced none, 20.4% experienced one type, 4.2% experienced two types, and 1.4% experienced three or more types. Health and lifestyle factors were observed, showing that 83.7% of participants had at least one chronic disease, 35.1% were current smokers, and 70.4% reported drinking behavior. Regarding social interaction, 64.9% of participants were classified as having rich social interaction. In terms of Ikigai, 60.9% of participants reported having Ikigai.

**TABLE 1:** Baseline demographic characteristics of participants (*n* = 504).

Variable	Category	*n*	%
Age	65–74	331	65.7
	≥ 75	173	34.3
Gender	Male	235	46.6
	Female	269	53.4
Subjective economic status	Economically disadvantaged	107	21.2
	Not economically disadvantaged	397	78.8
Depressive symptoms	Present	152	30.2
	Absent	352	69.8
Subjective cognitive complaints	Present	271	53.8
	Absent	233	46.2
Negative life events	None	373	74.0
	One type	103	20.4
	Two types	21	4.2
	≥ Three types	7	1.4
Chronic diseases	Have	422	83.7
	Not have	82	16.3
Smoking behavior	Current smoker	177	35.1
	Non‐smoker	327	64.9
Drinking behavior	Drinker	355	70.4
	Non‐drinker	149	29.6
Social interaction	Rich	327	64.9
	Not rich	177	35.1
Ikigai	Have	307	60.9
	Not have	197	39.1

Table 2 presents the prevalence of Ikigai and social interaction at follow‐up in 2020. At follow‐up, 52.2% of participants were classified as having rich social interaction, while 58.9% reported having Ikigai.

**TABLE 2:** Prevalence of Ikigai and social interaction at follow‐up in 2020 (*n* = 504).

Variable	Category	*n*	%
Social interaction	Rich	263	52.2
	Not rich	241	47.8
Ikigai	Have	297	58.9
	Not have	207	41.1

To assess potential attrition bias, baseline characteristics between participants lost to follow‐up (*n* = 334) and those included in the final analytic sample (*n* = 504) were compared. Those who were lost to follow‐up were older (≥ 75 years) and less likely to have rich social interaction and Ikigai at baseline (all *p* < 0.001). They were also more likely to present depressive symptoms (*p* = 0.007) and less likely to be current smokers and drinkers (both *p* < 0.001). In contrast, gender, subjective cognitive complaints, NLE, and chronic diseases did not significantly differ between groups. Detailed results are presented in Appendix S5.

**FIGURE 2:**
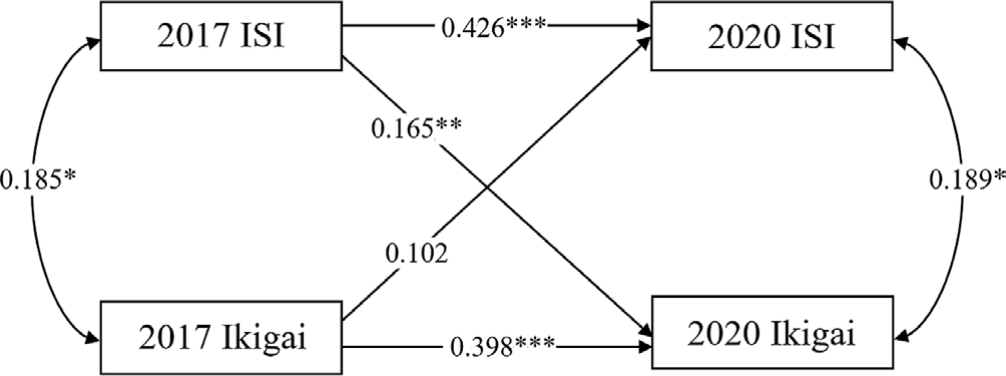
The CLPM of social interaction and Ikigai (2017–2020). Numbers represent standardized pathway coefficients. All coefficients are standardized. Straight lines with one‐way arrows represent structural pathways, and broken lines with two‐way arrows represent concurrent correlations. *χ* ^2^ = 20.376, DF = 18, *p* = 0.312, CFI = 0.949, TLI = 0.880, RMSEA = 0.016, 90% CI [0.000, 0.044], SRMR = 0.076. *p < 0.05, **p < 0.01, ***p < 0.001.

Figure 2 shows the fit indices for the CLPM, exhibiting an acceptable overall model fit, with *χ* ^2^ = 20.376, DF = 18, CFI = 0.949, TLI = 0.880, RMSEA = 0.016, and SRMR = 0.076. On the results of cross‐lagged effects, social interaction in 2017 significantly predicted Ikigai in 2020 (*β* = 0.165, *p* = 0.006), indicating that richer baseline social interaction was associated with an increased likelihood of possessing Ikigai in the subsequent measurement wave. The reverse pathway did not reach conventional statistical significance (*β* = 0.102, *p* = 0.060). Autoregressive effects were substantial for both social interaction (*β* = 0.426, *p* < 0.001) and Ikigai (*β* = 0.398, *p* < 0.001), indicating notable continuity of these constructs over time.

After further adjusting for changes in functional status and NLE during the follow‐up period, the association between baseline social interaction and Ikigai at follow‐up remained statistically significant, whereas the reverse pathway from baseline Ikigai to subsequent social interaction remained non‐significant. Detailed results of this sensitivity analysis are presented in Appendix S6.

The results of the supplementary binary logistic regression analyses are presented in Table 3. After baseline adjustment and controlling for multiple factors, social interaction in 2017 was significantly associated with Ikigai in 2020 (OR = 2.00, 95% CI = 1.29–3.12, *p* = 0.002). In addition, baseline Ikigai was significantly associated with social interaction in 2020 (OR = 1.55, 95% CI = 1.01–2.38, *p* = 0.044).

**TABLE 3:** Binary logistic regression analysis of factors associated with Ikigai and social interaction.

Variable	Ikigai in 2020		ISI in 2020	
	OR	95% CI	OR	95% CI
ISI in 2017	2.00**	1.29–3.12	6.56***	4.15–10.34
Ikigai in 2017	5.75***	3.76–8.79	1.55*	1.01–2.38
Age	1.06	0.68–1.66	1.65*	1.07–2.55
Gender	0.64	0.37–1.13	0.52*	0.30–0.90
Subjective economic status	0.51**	0.30–0.85	0.70	0.42–1.16
Depressive symptoms	0.66	0.41–1.06	0.93	0.58–1.50
Subjective cognitive complaints	0.91	0.59–1.41	0.91	0.59–1.39
NLE (one type)	0.88	0.53–1.48	1.51	0.91–2.52
NLE (two types)	1.05	0.36–3.11	1.90	0.66–5.52
NLE (≥ three types)	0.38	0.06–2.31	4.63	0.88–24.42
Chronic diseases	0.88	0.50–1.56	0.62	0.36–1.08
Smoking behavior	0.85	0.48–1.52	1.02	0.58–1.80
Drinking behavior	1.16	0.71–1.90	1.21	0.75–1.96

Abbreviations: CI, confidence interval; ISI, index of social interaction; NLE, negative life events; OR, odds ratio.

* *p* < 0.05.

** *p* < 0.01.

*** *p* < 0.001.

## 4 Discussion

Using a two‐wave binary‐outcome CLPM with WLSMV as the primary analytic framework, this study examined the temporal ordering between social interaction and Ikigai among community‐dwelling older adults. After accounting for baseline levels and relevant covariates, richer social interaction at baseline significantly predicted Ikigai 3 years later, whereas the reverse pathway from baseline Ikigai to subsequent social interaction did not reach conventional statistical significance under the present design and measurement conditions. Substantial autoregressive effects were observed for both constructs, indicating marked temporal stability. These findings provide directional and hypothesis‐generating evidence that social interaction precedes and facilitates the development of Ikigai over time.

Situated within an expanding body of longitudinal research linking social relationships (e.g., social participation, social support, loneliness, and social isolation) with mental health outcomes including depressive symptoms, anxiety, happiness, and life satisfaction, the present study extends by focusing on Ikigai as a culturally grounded indicator of positive and meaning‐oriented mental health [10, 15, 16, 17, 18, 19, 20, 21]. Whereas happiness and life satisfaction primarily reflect hedonic well‐being, including feeling good and evaluating one's life positively, Ikigai captures a more eudaimonic dimension of mental health, emphasizing perceptions of life worth living and a sense of purpose rather than simply the intensity of positive affect [1, 2, 3, 4, 6]. By examining Ikigai, this study extends prior longitudinal research that has primarily emphasized hedonic outcomes, and complements the relatively small number of CLPM analyses that have demonstrated the applicability of this framework, thereby broadening the conceptual scope of mental health outcomes considered in social relationship research.

Several interrelated mechanisms may underlie the observed longitudinal association between social interaction and subsequent Ikigai. First, social interaction may foster Ikigai through role accumulation and a sense of social contribution. Engagement in interpersonal exchanges and community activities provides older adults with opportunities to feel useful, needed, and socially valued, which are core elements of Ikigai as a meaning‐oriented construct [22, 24, 25]. Ikigai, as conceptualized in prior literature, emphasizes sustained role fulfillment and perceived purpose rather than transient positive affect [3]. Second, social interaction may indirectly support Ikigai by buffering psychological distress through emotional and instrumental support. Longitudinal evidence indicates that social relationships mitigate depressive symptoms and anxiety by enhancing emotion regulation and reducing perceived stress [10, 18]. Although this pathway primarily explains reductions in outcomes related to distress, lower psychological distress may create conditions that enable the maintenance of Ikigai over time.

Compared with conventional bidirectional logistic regressions, the CLPM retained the association from social interaction to Ikigai after accounting for temporal stability and synchronous correlations, underscoring its incremental value for examining temporal ordering under dichotomous measurement [39]. By modeling both variables simultaneously, the CLPM helps distinguish temporal precedence from cross‐sectional correlation, even when binary indicators are used [27, 28, 44]. The direction from social interaction to Ikigai is consistent with social resource and role‐accumulation perspectives in aging, whereby engagement and support provide older adults with opportunities to develop and sustain Ikigai, as suggested by prior cross‐sectional research linking social participation and support to Ikigai [22, 24, 25]. Extending prior longitudinal evidence [16], our cohort design using a cross‐lagged approach directly tested temporal ordering and demonstrated that richer baseline social interaction predicted subsequent Ikigai after accounting for both stability and concurrent covariation. Sensitivity analyses incorporating changes in IADL and follow‐up negative life events indicated that the main pathway from social interaction to subsequent Ikigai remained statistically significant, suggesting relative robustness to time‐varying influences during the follow‐up period.

Conversely, the pathway from baseline Ikigai to subsequent social interaction did not reach conventional statistical significance in the CLPM (*p* = 0.060), although baseline‐adjusted logistic regression analyses indicated a statistically significant association. This discrepancy likely reflects fundamental differences between the two analytical approaches. Conventional regression models estimate the total association between baseline Ikigai and follow‐up social interaction, which may partly capture stable between‐person differences and concurrent correlations at baseline. In contrast, the CLPM explicitly accounts for the strong temporal stability of both constructs and their within‐wave associations, thereby isolating the prospective effect of Ikigai on changes in social interaction over time. As a result, CLPM estimates tend to be more conservative and may yield attenuated effect sizes. From this perspective, the weaker pathway from baseline Ikigai to subsequent social interaction observed in the CLPM should not be interpreted as evidence against a reciprocal relationship per se, but rather as reflecting both a more stringent modeling framework and the conditional nature of this pathway. Nevertheless, although the cross‐lagged framework allows examination of temporal ordering, the findings should be interpreted as hypothesis‐generating rather than definitive causal evidence, particularly given the two‐wave design and dichotomous measurement.

Another possible explanation relates to the timing of the follow‐up survey during the COVID‐19 pandemic. In the present study, the proportion of participants classified as having rich social interaction decreased from 64.9% in 2017 to 52.2% in 2020. This decline may reflect the lingering effects of pandemic‐related social restrictions among older adults. In Japan, social interaction in later life is often maintained through face‐to‐face activities, neighborhood engagement, and participation in local community organizations. Consequently, even older adults with a strong sense of Ikigai may have encountered limited opportunities to translate their psychological resources into actual social interaction during this period. Previous research among community‐dwelling older adults in Japan has shown that the COVID‐19 pandemic substantially disrupted daily activities, social participation, and interpersonal contact, with potential consequences for social connectedness and psychological health [45]. Therefore, pandemic‐related constraints may have attenuated the prospective association between baseline Ikigai and subsequent social interaction, contributing to the non‐significant cross‐lagged pathway observed in the present study.

In addition, this study demonstrates the practical feasibility of implementing and reporting a binary‐outcome CLPM in community‐based aging research. We describe a clear workflow, including defining how variables are dichotomized, modeling with WLSMV and probit links [27, 46], and reporting STDYX‐standardized coefficients and fit indices [47]. We also compare the results with those from conventional regressions to facilitate replicability and comparability across applications.

Interpreted as directional evidence, our findings suggest that expanding opportunities for meaningful, regular interaction in daily life may help promote Ikigai in later life. At the same time, the weaker reverse pathway observed under the cross‐lagged modeling framework suggests that Ikigai alone may not consistently translate into sustained interaction over time without supportive social and environmental conditions.

The limitations of this study are noteworthy. First, the sample was recruited from a specific geographic area in Japan, which may limit the generalizability of the findings. Second, Ikigai was operationalized as a binary indicator, and social interaction was treated dichotomously. Although the cross‐lagged association remained statistically significant (*p* < 0.01), dichotomization may have attenuated effect sizes and limited the ability to detect dose–response relationships. Future studies using continuous or multi‐item measures are recommended to further examine the robustness and potential nonlinear nature of this association. Third, the relatively small sample size may affect the stability and precision of the cross‐lagged estimates, even though missing data were addressed using multiple imputation. Fourth, attrition represents another limitation of this study. A non‐negligible proportion of participants were lost to follow‐up, and attrition was not completely random. Those lost differed significantly from the analytic sample in age, depressive symptoms, baseline social interaction, baseline Ikigai, and smoking and drinking behavior. The selective loss of individuals with both poorer social interaction and lack of Ikigai is particularly noteworthy, as these characteristics are directly related to the main constructs of interest. This attrition pattern may have resulted in an analytic sample that was socially more active and more likely to report Ikigai, potentially leading to conservative estimates of the associations between social interaction and Ikigai. Accordingly, the findings should be interpreted with caution, especially regarding their generalizability to older adults with limited social engagement and lower levels of Ikigai. Fifth, the follow‐up survey was conducted shortly after the first COVID‐19 state of emergency in Japan. Pandemic‐related restrictions and behavioral changes may have influenced both patterns of social interaction and perceptions of Ikigai, independent of longer‐term psychosocial processes. Although prevalence data in 2020 were reported and sensitivity analyses were conducted, the specific impact of COVID‐19‐related factors could not be disentangled within the present study design. Finally, although methodological literature suggests that two‐wave designs can yield informative results when CLPMs are carefully specified [40, 41], models with only two waves are limited in their ability to separate within‐ and between‐person variance, evaluate stationarity, and ensure stable estimates across samples. Therefore, for observing more stable lag effects and subtle bidirectional changes, future research is recommended to use larger sample sizes, more waves, richer measures, and varied lags to strengthen temporal inferences.

## 5 Conclusions

This study demonstrates that the binary‐outcome CLPM is both feasible and informative for examining temporal ordering under dichotomous measurement in community aging research. Our findings provide stronger support for the pathway from baseline social interaction to subsequent Ikigai, whereas the reverse pathway did not reach conventional statistical significance in the CLPM after accounting for temporal stability. These results underscore the methodological value of the CLPM framework in distinguishing between total associations and prospective effects on change over time. Beyond methodological advancement, the findings highlight the potential community relevance of fostering social interaction as a pathway to sustaining Ikigai in later life.

## Author Contributions

Y.P. conceptualized and designed the study, conducted data cleaning, formal analysis, and modeling, and prepared the original manuscript draft. A.Y. contributed to the study conception, participated in drafting, and revised the manuscript. C.N. contributed to the study conception, participated in drafting, and provided critical revisions. T.M. and K.W.M. reviewed the manuscript and provided constructive feedback. Y.S. and A.K. contributed to data investigation, collection, and curation. T.A. was the primary investigator of the original cohort underlying this study, supervised the project, acquired funding, and provided overall project administration. All authors contributed to the interpretation of the results and approved the final version of the manuscript.

## Funding

This work was supported by the Japan Science and Technology Agency (JST) through the Support for Pioneering Research Initiated by the Next Generation (SPRING) program (Grant Number JPMJSP2124).

## Ethics Statement

This study was approved by the Ethics Review Committee of the University of Tsukuba (authorization No. 1331‐5). All processes in this study were conducted in accordance with the Declaration of Helsinki.

## Consent

This study did not involve any patients. Since the data used were anonymous, the Ethics Review Committee of the University of Tsukuba waived written informed consent in accordance with the rules of the Japanese Ethical Guidelines for Medical and Health Research Involving Human Subjects.

## Conflicts of Interest

The authors declare no conflicts of interest.

## Supporting information


**Appendix S1:** Distribution of ISI Scores in 2017.
**Appendix S2:** Distribution of ISI Scores in 2020.
**Appendix S3:** Index of Social Interaction (ISI).
**Appendix S4:** Classification of Covariates.
**Appendix S5:** Baseline Comparison Between Participants Included in the Analysis and Those Lost to Follow‐Up.
**Appendix S6:** Sensitivity Analysis Including Changes in IADL and Follow‐up NLE.

## Data Availability

The data that support the findings of this study are available from the corresponding author upon reasonable request.
