# Supplementary material for: Beyond Continuity: A Binary‐Outcome Cross‐Lagged Panel Model of Ikigai and Social Interaction Among Community‐Dwelling Older Adults
Source: Geriatr Gerontol Int. 2026 Aug 5;26(8):e70755. doi: 10.1111/ggi.70755 (PMC13441515; doi:10.1111/ggi.70755)
Supplement: Supplementary file 1 [file GGI-26-0-s001.docx]

**Supplementary Material**

**Title:**

Beyond Continuity: A Binary-Outcome Cross-Lagged Panel Model of Ikigai and Social Interaction among Community-Dwelling Older Adults

**Contents:**

Appendix 1. Distribution of ISI Scores in 2017

Appendix 2. Distribution of ISI Scores in 2020

Appendix 3. Index of Social Interaction (ISI)

Appendix 4. Classification of Covariates

Appendix 5. Baseline Comparison Between Participants Included in the Analysis and Those Lost to Follow-Up

Appendix 6. Sensitivity Analysis Including Changes in IADL and Follow-up NLE

References (for Supplementary Material)

**Appendix 1. Distribution of ISI Scores in 2017**

ISI scores ranged from 0 to 18. The distribution was highly skewed toward the upper end, with most participants scoring 17 or 18.

**Appendix 2. Distribution of ISI Scores in 2020**

ISI scores ranged from 0 to 18. A similar ceiling effect was observed in 2020, with the majority of participants scoring 17 or 18.

**Appendix 3. Index of Social Interaction (ISI)**

| Question | Answer | | | |
| --- | --- | --- | --- | --- |
| 1) Independence |  |  |  |  |
| Having a motivation to live an active lifestyle | Always | Often | Sometimes | Rarely |
| Taking an active approach towards one’s life | Always | Often | Sometimes | Rarely |
| Being motivated to live a healthy life | Always | Often | Sometimes | Rarely |
| Having a regular or routine lifestyle | Always | Often | Sometimes | Rarely |
| 2) Social curiosity |  |  |  |  |
| Reading newspapers | Always | Often | Sometimes | Rarely |
| Reading books | Always | Often | Sometimes | Rarely |
| Trying to use new equipment | Always | Often | Sometimes | Rarely |
| Having a hobby | Always | Often | Sometimes | Rarely |
| Having a feeling of importance | Always | Often | Sometimes | Rarely |
| 3) Interaction |  |  |  |  |
| Communication within the family | Always | Often | Sometimes | Rarely |
| Communication with non-family persons | Always | Often | Sometimes | Rarely |
| Interaction with non-family persons | Always | Often | Sometimes | Rarely |
| 4) Participation in society |  |  |  |  |
| Participation in social groups | Always | Often | Sometimes | Rarely |
| Watching television | Always | Often | Sometimes | Rarely |
| Participation in neighborhood affairs | Always | Often | Sometimes | Rarely |
| Having an active role in society | Always | Often | Sometimes | Rarely |
| 5) Feelings of safety |  |  |  |  |
| Having counsel | Always | Often | Sometimes | Rarely |
| Having someone to give support in an emergency | Always | Often | Sometimes | Rarely |

Each item is scored as one point for a positive response (always/often/sometimes) and zero for a negative response (rarely). Missing values are replaced with the mean score of the remaining items. The total score ranges from 0 to 18.

**Appendix 4. Classification of Covariates**

Subjective economic status was assessed by the question, “How do you feel about your current living conditions in terms of financial situation?” Depressive symptoms were assessed by two questions, “During the past month, have you felt down, depressed, or hopeless?” and “During the past month, have you felt little interest or enjoyment in doing things?” Subjective cognitive complaints were assessed by two questions, “Do you feel that you forget things more often?” and “Do you sometimes have difficulty remembering today’s date?” NLE included events experienced by participants or their family members within the past year, such as moving residence, resigning or retiring, changing jobs, being hospitalized, separating or divorcing, experiencing death, encountering financial troubles, and facing interpersonal conflicts. The classification of NLE referenced the social readjustment rating scale (1–2) and the list of threatening experiences (3–4), which enumerate past-year events as potential stressors. Chronic diseases included physician-diagnosed conditions, including diabetes, stroke, hypertension, hyperlipidemia, heart diseases, cancers, kidney diseases, stomach diseases, respiratory diseases, immune diseases, muscle diseases, ear diseases, eye diseases, Parkinson’s disease, dementia, and depression.

Age was categorized as 65-74 years and ≥ 75 years. Gender was categorized as male and female. Subjective economic status was categorized as economically disadvantaged (including very difficult and somewhat difficult) and not economically disadvantaged (including average, somewhat comfortable, and very comfortable). Depressive symptoms and subjective cognitive complaints were categorized as present if participants responded affirmatively to at least one of the two items and as absent if they responded negatively to both items. The NLE was categorized as not experienced, experienced one type, experienced two types, and experienced three or more types. Chronic diseases were categorized as having at least one of the mentioned diseases or no chronic diseases. Smoking behavior was categorized as current smokers (including those who smoke almost every day or sometimes) and non-smokers (including those who never smoke or used to smoke but quit). Drinking behavior was categorized as drinkers (including those who drink almost every day, sometimes, or rarely) and non-drinkers. Social interaction in 2017 was categorized as rich and not rich. Ikigai in 2017 was categorized as having and not having it.

**Appendix 5. Baseline Comparison Between Participants Included in the Analysis and Those Lost to Follow-Up**

| Variable | Category | Analyzed (n = 504) |  | Lost to follow-up (n = 334) | *p* |
| --- | --- | --- | --- | --- | --- |
|  |  | n (%) |  | n (%) |  |
| Age | 65-74 | 331 (65.7) |  | 151 (45.2) | < .001 |
|  | ≥75 | 173 (34.3) |  | 183 (54.8) |  |
| Gender | Male | 235 (46.6) |  | 153 (45.8) | .816 |
|  | Female | 269 (53.4) |  | 181 (54.2) |  |
| Subjective economic status | Economically disadvantaged | 107 (21.2) |  | 86 (25.7) | .128 |
|  | Not economically disadvantaged | 397 (78.8) |  | 248 (74.3) |  |
| Depressive symptoms | Present | 152 (30.2) |  | 131 (39.2) | .007 |
|  | Absent | 352 (69.8) |  | 203 (60.8) |  |
| Subjective cognitive complaints | Present | 271 (53.8) |  | 199 (59.6) | .097 |
|  | Absent | 233 (46.2) |  | 135 (40.4) |  |
| Negative life events | None | 373 (74.0) |  | 239 (71.6) | .400 |
|  | One type | 103 (20.4) |  | 67 (20.0) |  |
|  | Two types | 21 (4.2) |  | 19 (5.7) |  |
|  | ≥ Three types | 7 (1.4) |  | 9 (2.7) |  |
| Chronic diseases | Have | 422 (83.7) |  | 292 (87.4) | .169 |
|  | Not have | 82 (16.3) |  | 42 (12.6) |  |
| Smoking behavior | Current smoker | 177 (35.1) |  | 38 (11.4) | < .001 |
|  | Non-smoker | 327 (64.9) |  | 296 (88.6) |  |
| Drinking behavior | Drinker | 355 (70.4) |  | 197 (59.0) | < .001 |
|  | Non-drinker | 149 (29.6) |  | 137 (41.0) |  |
| Social interaction | Rich | 327 (64.9) |  | 115 (34.4) | < .001 |
|  | Not rich | 177 (35.1) |  | 219 (65.6) |  |
| Ikigai | Have | 307 (60.9) |  | 160 (52.1) | < .001 |
|  | Not have | 197 (39.1) |  | 174 (47.9) |  |

**Appendix 6. Sensitivity Analysis Including Changes in IADL and Follow-up NLE**

| Path | *β* | SE | *p* |
| --- | --- | --- | --- |
| ISI 2017 → Ikigai 2020 | 0.374 | 0.137 | .006 |
| Ikigai 2017 → ISI 2020 | 0.215 | 0.138 | .118 |
| ISI 2017 → ISI 2020 | 0.979 | 0.104 | < .001 |
| Ikigai 2017 → Ikigai 2020 | 0.925 | 0.112 | < .001 |
| IADL → ISI 2020 | -0.512 | 0.170 | .003 |
| IADL → Ikigai 2020 | -0.379 | 0.152 | .013 |
| NLE → ISI 2020 | 0.153 | 0.101 | .129 |
| NLE → Ikigai 2020 | 0.145 | 0.098 | .142 |

Functional status was assessed using six instrumental activities of daily living (IADL) items: using public transportation independently (bus or train), including driving a private car; shopping independently for food and daily necessities; preparing meals independently; paying bills independently; managing deposits and withdrawals from savings accounts independently; and completing administrative paperwork (e.g., pension-related documents or forms submitted to government offices or hospitals). These six items were based on the Tokyo Metropolitan Institute of Gerontology Index of Competence (TMIG-IC), including five instrumental self-maintenance items plus one item from the intellectual activity domain, namely completing administrative paperwork (5). Each item had three response options: able to do and currently doing; able to do but not currently doing; and unable to do. Responses indicating inability were coded as 1, whereas the other responses (able regardless of current performance) were coded as 0. A total IADL score was calculated by summing the six items, yielding a possible range from 0 to 6, with higher scores indicating greater functional limitation. Changes in IADL were calculated as the difference between the follow-up and baseline total scores. If the IADL score was missing at either wave, the change score was coded as missing. NLE included events experienced by participants or their family members within the past year, such as moving residence, resigning or retiring, changing jobs, being hospitalized, separating or divorcing, experiencing death, encountering financial troubles, and facing interpersonal conflicts. Because NLE was assessed with reference to the past year, NLE measured at follow-up was treated as reflecting events occurring during the follow-up period.

The distribution of changes in IADL was as follows: improvement (ΔIADL < 0), n = 24 (4.8%); no change (ΔIADL = 0), n = 397 (78.8%); deterioration (ΔIADL > 0), n = 48 (9.5%); and missing data, n = 35 (6.9%). The distribution of follow-up NLE categories was as follows: not experienced, n = 365 (72.4%); experienced one type, n = 109 (21.6%); experienced two types, n = 24 (4.8%); and experienced three or more types, n = 6 (1.2%).

All coefficients are standardized. SE indicates standard error. Model fit indices for the sensitivity analysis (estimated using WLSMV) were χ² = 24.989, DF = 22, *p* = .298, CFI = 0.932, TLI = 0.846, RMSEA =0.017, 90% CI [0.000, 0.044], SRMR = 0.070.

**References:**

(1) Hobson CJ, Delunas L. National norms and life-event frequencies for the revised Social Readjustment Rating Scale. Int J Stress Manag. 2001;8(4):299–314. doi:10.1023/A:1017565632657

(2) Wallace D, Cooper NR, Sel A, Russo R. The social readjustment rating scale: Updated and modernised. PLoS One. 2023;18(12):e0295943. doi:10.1371/journal.pone.0295943

(3) Brugha T, Bebbington P, Tennant C, Hurry J. The List of Threatening Experiences: A subset of 12 life event categories with considerable long-term contextual threat. Psychol Med. 1985;15(1):189–194. doi:10.1017/S003329170002105X

(4) Rosmalen JGM, Bos EH, de Jonge P. Validation of the Long-Term Difficulties Inventory (LDI) and the List of Threatening Experiences (LTE) as measures of stress in epidemiological population-based cohort studies. Psychol Med. 2012;42(12):2599–2608. doi:10.1017/S0033291712000608

(5) Koyano W, Shibata H, Nakazato K, Haga H, Suyama Y. Measurement of competence: Reliability and validity of the TMIG Index of Competence. Arch Gerontol Geriatr. 1991;13:103-116. doi:10.1016/0167-4943(91)90053-S.
